# Perforation of Double-Spaced Aluminum Plates by Reactive Projectiles with Different Densities

**DOI:** 10.3390/ma14051229

**Published:** 2021-03-05

**Authors:** Hao Zhang, Haifu Wang, Qingbo Yu, Yuanfeng Zheng, Guancheng Lu, Chao Ge

**Affiliations:** State Key Laboratory of Explosion Science and Technology, Beijing Institute of Technology, Beijing 100081, China; haozhang@bit.edu.cn (H.Z.); wanghf@bit.edu.cn (H.W.); yuqb@bit.edu.cn (Q.Y.); zhengyf@bit.edu.cn (Y.Z.); 3120195177@bit.edu.cn (G.L.)

**Keywords:** perforation, reactive projectile, density, double-spaced plates, damage behavior

## Abstract

Perforation behavior of 3 mm/3 mm double-spaced aluminum plates by PTFE/Al/W (Polytetrafluoroethylene/Aluminum/Tungsten) reactive projectiles with densities ranging from 2.27 to 7.80 g/cm^3^ was studied experimentally and theoretically. Ballistic experiments show that the failure mode of the front plate transforms from petalling failure to plugging failure as projectile density increases. Theoretical prediction of the critical velocities for the reactive projectiles perforating the double-spaced plates is proposed, which is consistent with the experimental results and well represents the perforation performance of the projectiles. Dimensionless formulae for estimating the perforation diameter and deflection height of the front plates are obtained through dimensional analysis, indicating material density and strength are dominant factors to determine the perforation size. High-speed video sequences of the perforation process demonstrate that high-density reactive projectiles make greater damage to the rear plates because of the generation of projectile debris streams. Specifically, the maximum spray angle of the debris streams and the crater number in the debris concentration area of the rear plate both increase with the projectile density and initial velocity.

## 1. Introduction

Reactive projectiles incorporate the strength and energy advantages of novel reactive materials. When the impact velocity reaches a certain value, reactive projectiles would produce an impact-induced chemical reaction, causing unique damage modes [1,2,3]. Compared with traditional inert metal projectiles, reactive projectiles demonstrate enhanced perforation diameter and damage effects to various targets.

Over the past decade, reactive materials have been extensively studied, and a series of potential engineering applications have been obtained due to their unique characteristics. A specific class of reactive materials is active metal particle filled polymer-matrix composites, such as PTFE/Al (Polytetrafluoroethylene/Aluminum), PTFE/Ti (Polytetrafluoroethylene/Titanium), etc. Based on the basic composition, high density metal particles, such as tungsten powders are often introduced to improve the density and mechanical strength of reactive materials. The relating research includes mechanical properties [4,5,6,7], impact response [8,9,10], and energy release characteristics [11,12] of the reactive materials. Research have been conducted towards potential engineering applications, mainly focusing on the damage effects of reactive projectiles, fragments and jets to the target bodies and behind-target effects [13,14,15,16,17,18].

During the interaction between projectiles and targets, properties of each part and impact conditions are all relevant factors deciding the eventual response of projectiles and targets. Especially for the reactive projectiles, the kinetic impact and chemical energy release are combined during the perforation process [19,20], which brings a challenge in analyzing projectile target interaction. Thus, the perforation behavior and collision mechanism of reactive projectiles are important to be systematically studied and characterized.

Based on the above consideration, this paper mainly focuses on the perforation of double-spaced aluminum plates by reactive projectiles of different densities. Firstly, the perforation experiment on double-spaced thin aluminum plates is presented. Then, perforation performance of the reactive projectiles is discussed, and the perforation limits are theoretically analyzed. Subsequently, the perforation characteristics of the front plates by reactive projectiles of different densities are discussed. The dimensionless relationship for the perforation diameter and the deflection height of the front plates are obtained through dimensional analysis. Finally, the perforating processes of metallic double-spaced plates by different densities reactive projectiles are compared, and the damage behavior of rear plates is analyzed.

## 2. Perforation Experiments

### 2.1. Preparation of Reactive Projectiles

**Figure 1:**
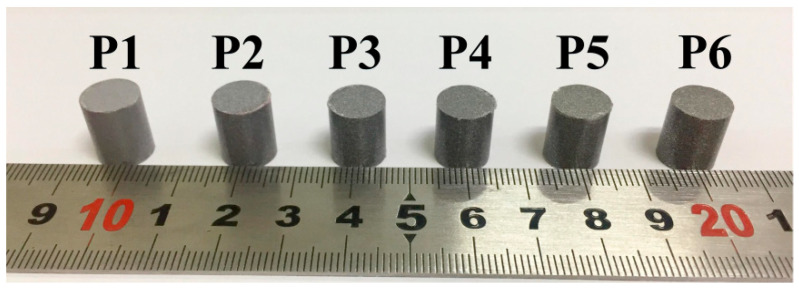
Six types of prepared reactive projectiles.

In this study, PTFE/Al/W reactive projectiles of six different densities were prepared. The components, densities and masses of the six types of reactive projectiles are listed in Table 1. Preparation process of the projectiles includes three steps. Firstly, powders of PTFE, Al, and W were weighed and mixed uniformly according to compositions of the corresponding projectile type, where PTFE/Al is a stoichiometric mixture with the ratio of 73.5:26.5. The average diameters of PTFE, Al and W powders are 100, 44, and 44 μm, respectively. The dry mixing time of the powders for each composition was up to 24 h to ensure the reactive powders were mixed uniformly. Then, the uniformly mixed powders were pressed into a steel mold at the pressure of 200 MPa, and the dwell time was approximately 10 min to form compact and homogenized projectile samples. Finally, the pressed samples underwent a sintering cycle in a furnace under the protection of argon atmosphere to avoid oxidation. Temperature history of the sintering process was described in the previously published paper [19]. The diameter and height of the projectiles are 10 and 12 mm, respectively. The six types of prepared reactive projectiles used for perforation experiments are shown in Figure 1. Moreover, the size and actual density of the tested reactive projectiles are listed in Table 2.

### 2.2. Experiment Method

**Figure 2:**
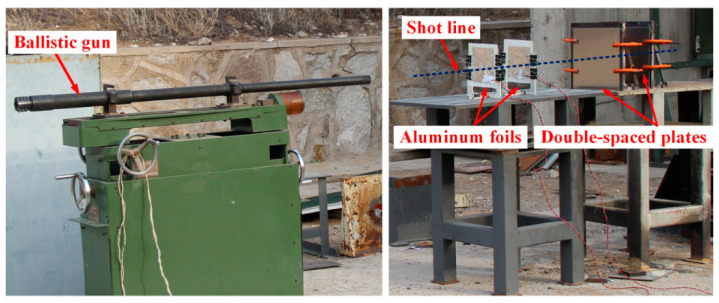
Schematic of the experimental setup.

**Figure 3:**
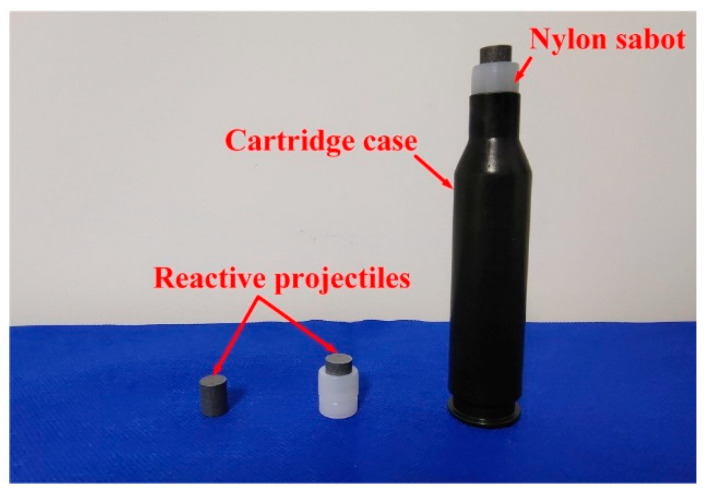
Assembled reactive projectile.

Perforation experiments of reactive projectiles of six different densities were conducted by employing a 14.5 mm caliber ballistic gun system, and schematic of the experimental setup is shown in Figure 2. To be launched by the 14.5 mm caliber ballistic gun, reactive projectiles were firstly packaged within nylon sabots. Figure 3 presents the assembled reactive projectile. Launching velocities ranged from 604 to 1063 m/s, and were adjusted by the propellant powder amount. Impact velocities of the projectiles were measured by a velocity measuring system, which was composed of two aluminum foils and an electronic timer. The distance between the two aluminum foils was 300 mm. Behind the system, two 3 mm thick 2024-T3 aluminum plates were mounted by target holders, the transverse and longitudinal dimensions of the plates were both 310 mm, and the distance between two aluminum plates was 400 mm. To ensure that the reactive projectiles could normally impact the targets, the distance between the gun muzzle and the front aluminum plate was 2.5 m. At a certain distance away from the area where the projectile and targets interact, a high-speed camera was set for the purpose of observation of the impact process. The frame rate and exposure time of the high-speed camera was set to 12,000 fps and 83 μs, respectively.

## 3. Experimental Results

**Figure 4:**
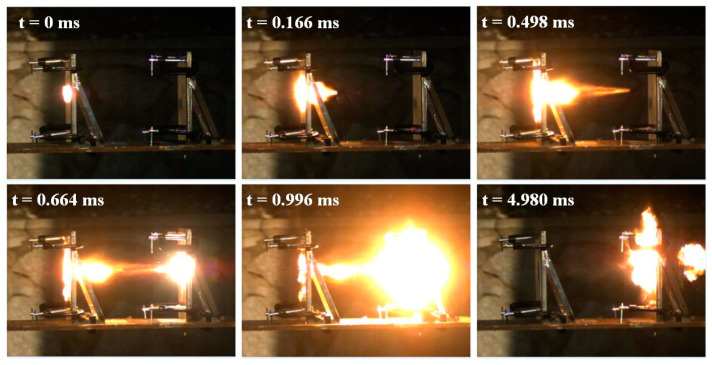
Typical high-speed video frames of P2 reactive projectile perforating double-spaced aluminum plates at 792 m/s.

Figure 4 captures the typical high-speed video frames of reactive projectile perforating double-spaced aluminum plates at about 800 m/s. During the initial stage from 0 to 0.166 ms, the reactive projectile initiated a deflagration reaction upon it impacting the front plate, and part of the energy was released during perforating the front plate as the first and second images show. Following this stage, the activated residual projectile continued to fly and impact the rear plate, inducing a violent reaction and producing a luminous spherical flame region around the plate which is visible in the fifth image. Until t =4.980 ms, the residual projectile experienced a violent reaction, and the flame around the rear plate gradually extinguished.

**Figure 5:**
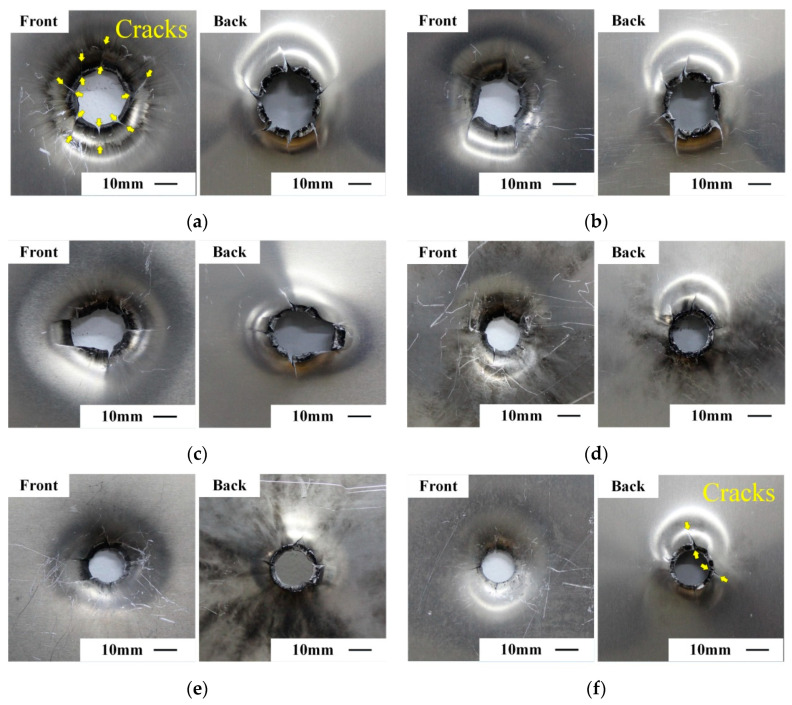
Front and back views of front aluminum plates impacted by reactive projectiles with different densities at the speed of ~800 m/s. (**a**) P1 at 798 m/s, (**b**) P2 at 792 m/s, (**c**) P3 at 762 m/s, (**d**) P4 at 790 m/s, (**e**) P5 at 824 m/s, (**f**) P6 at 817 m/s.

Perforation holes in the front plates by six types of reactive projectiles at the speed of about 800 m/s are shown in Figure 5. By comparison, the perforation diameter reduces with increasing projectile density, and the change of failure mechanisms of the front plates can also be observed. For the low-density reactive projectiles, as shown in Figure 5a–c, the failure mode is petalling failure. Obvious bulge and petalling deflection exist in these plates. While for the high-density reactive projectiles in Figure 5d–f, because of the improvement of projectile strength and penetration capability, the failure mode changes into typical plugging failure. In addition, cracks are found around the perforation hole, and the number of cracks gradually decreased as the projectile density increased. Specifically, there were seven clear cracks around the perforation hole when the projectile density was 2.27 g/cm^3^ (Type P1) as shown in Figure 5a, but only two short cracks as the projectile density increased to 7.80 g/cm^3^ (Type P6), see Figure 5f. This is another signal indicating the failure mode transformation of the plate.

**Figure 6:**
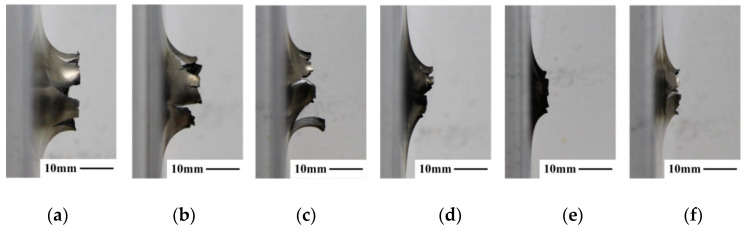
Side view of front aluminum plates deflection heights impacted by reactive projectiles with different densities at the speed of ~800 m/s. (**a**) P1 at 798 m/s, (**b**) P2 at 792 m/s, (**c**) P3 at 762 m/s, (**d**) P4 at 790 m/s, (**e**) P5 at 824 m/s, (**f**) P6 at 817 m/s.

Observing from the side view, deflection around the perforations on the front plates by projectiles of different densities is demonstrated in Figure 6. It can be found that under the same impact condition, the deflection heights of the front aluminum plates reduce as the projectile density increases. Higher deflection heights occur in the plates for low-density projectiles, due to the impact-induced intense deflagration during the perforation process, resulting in rupturing effect in the plates. However, for the high-density projectiles, non-significant deflagration reacted during perforation, and no obvious rupturing effect was found in the front plates.

**Figure 7:**
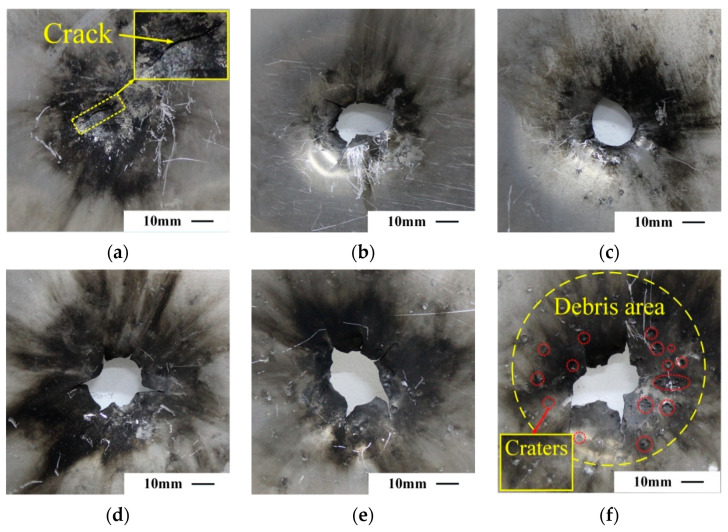
Front view of rear aluminum plates impacted by reactive material projectiles with different densities at a common speed of ~800 m/s. (**a**) P1 at 798 m/s, (**b**) P2 at 792 m/s, (**c**) P3 at 762 m/s, (**d**) P4 at 790 m/s, (**e**) P5 at 824 m/s, (**f**) P6 at 817 m/s.

Figure 7 shows the front perspective of the rear aluminum plates impacted by reactive projectiles of different densities at the speed of about 800 m/s. As arrowed in Figure 7a, the P1 projectile failed to perforate the rear plate, and only formed a crack in the rear plate, which demonstrates that the reactive projectiles without tungsten show weak perforation performance. Analyzing from the damage morphology of the plates, the destruction modes resulting from the reactive projectiles of various densities are different. The low-density projectile impacts the rear plate as an intact residual penetrator after perforating the front plate, and only causes a perforation hole in the rear plate, as shown in Figure 7b,c. Whereas, with density increasing, the reactive projectile not only produces a main perforation hole in the rear plate, but also forms a cratering and pitting circular area around the perforated hole, as shown in Figure 7e,f. That is the projectile debris area which was hit by the fragmented reactive projectile stream. As the debris stream collided with the metallic plate, a violent deflagration reaction was induced and left a large amount of carbon products on the rear plate surface. Meanwhile, with the increase in projectile density, the brittleness, kinetic energy, and perforation capability of the projectiles were enhanced, which resulted in the reactive projectiles being more fragmented after perforating the front plate, the debris streams spreading more broadly, and therefore the perforation diameter, debris area, and crater numbers in the rear plate all significantly increased.

Summary of the projectile initial velocity, perforation diameter in each plate, and deflection height of the front plates are listed in Table 3. Meanwhile, due to the irregular shape of the perforation holes in the rear plates, the perforated areas were equivalent to circular for characterization. Under the same initial velocities, the perforation diameter and the deflection height of the front plates decreased with the increase in projectile density. On the contrary, perforation area in the rear plates enlarged as the projectile density grew. It should be noted that when subjected to the low-density reactive projectiles (Type P1, P2) with the same initial velocities, the rear plates failed to be perforated. This demonstrates that the PTFE/Al/W reactive projectiles of different densities occupy different perforation capabilities.

## 4. Discussion

### 4.1. Perforation Limit of Reactive Projectiles

To investigate the perforation performance of the reactive projectiles on double-spaced aluminum plates, the ballistic limit velocities of the reactive projectiles impacting the front aluminum plate are calculated according to the empirical formula based on THOR theory firstly [19]:

(1) $$v_{b}=1855.7\cdot {\left(h_{t}\cdot A\right)}^{0.4143}m_{p}{}^{-0.5549}$$

(2) $$v_{r}=\frac{m_{P}}{m_{P}+m_{t}}{\left(v_{0}^{2}-v_{b}^{2}\right)}^{\frac{1}{2}}$$

where *v_b_* (m/s) is the ballistic limit velocity of the projectile, *h_t_* (cm) is the thickness of the target, *A* (cm^2^) is the incident area of the projectile, and *m_p_* (g) is the projectile mass. Furthermore, the residual velocity of the reactive projectiles after perforating the front aluminum plate *v_r_* (m/s) can be calculated according to the energy conservation, as shown in the above Equation (2) [21], where *m_t_* (g) is the plate plug mass, *v*_0_ (m/s) is the initial velocity of the projectile.

**Figure 8:**
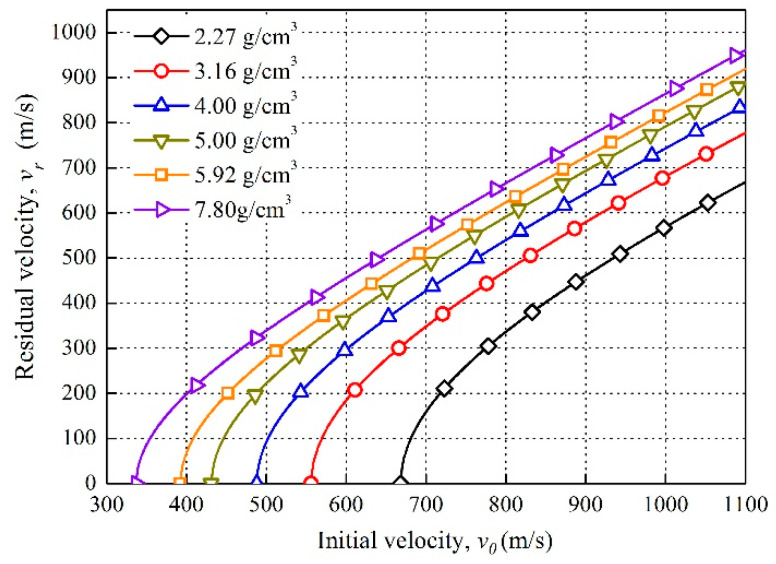
Residual velocity versus initial velocity for reactive projectiles perforating 3 mm aluminum plate.

According to Equation (1), Figure 8 depicts the initial velocity and corresponding residual velocity for reactive projectiles of different densities after perforating the front plate. As it can be seen, the ballistic limits of the reactive projectiles with various densities are different, and it decreases from 668 to 337 m/s with the increase in projectile density from 2.27 to 7.8 g/cm^3^. Moreover, under the identical initial velocity, the residual velocity of the reactive projectiles increase with the projectile density, which illustrates high-density projectiles have less velocity loss after perforating the front plate.

The kinetic energy of the residual penetrators (including the projectile and plate plug) *E_r_* (J) is shown in Equation (3), where *ρ_t_* (g/cm^3^) is the aluminum plate density. The diameter of the plug is assumed to be equal to the projectile’s diameter, and the plug velocity is consistent with the residual velocity of the projectile after perforating the front plate.

(3) $$E_{r}=\frac{1}{2}m_{p}v_{r}{}^{2}+\frac{1}{2}\rho_{t}Ah_{t}v_{r}{}^{2}$$

(4) $$E_{c}=kAh_{t}\sigma_{t}$$

**Figure 9:**
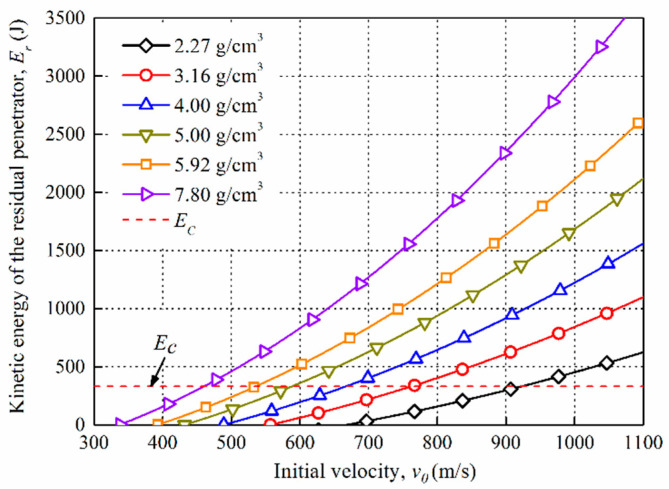
Kinetic energy of the residual penetrator versus initial velocity.

The residual penetrators continue to perforate the rear plate after perforating through the first plate. Here, *E_c_* (J) is defined as the critical energy to perforate the second plate [22], as shown in Equation (4), where *k* is the coefficient of the target material, for aluminum the value is 3 [22], *A* (cm^2^) is the encounter area for the projectile and the aluminum plate, *h_t_* is the thickness of the target, and the strength of the aluminum plate *σ_t_* is 470 Mpa [22]. Figure 9 illustrates the relationship between the kinetic energy of the residual penetrators *E_r_* (J) after perforating the front plate and the initial velocity of the projectiles *v*_0_ (m/s). *E_c_* is the critical energy required to perforate the rear plate. It can be seen that the kinetic energy of residual penetrator increases as projectile density and initial velocity increase, and this indicates that a high-density projectile brings better perforation performance.

Furthermore, the critical perforation velocity for the projectiles to perforate the double-spaced plates *v_ci_* (m/s) is obtained by substituting Equations (3) and (4) into Equation (2), as described in Equation (5):

(5) $$v_{ci}={\left[v_{b}^{2}+\frac{2E_{c}\left(m_{P}+m_{t}\right)}{m_{P}{}^{2}}\right]}^{\frac{1}{2}}$$

**Figure 10:**
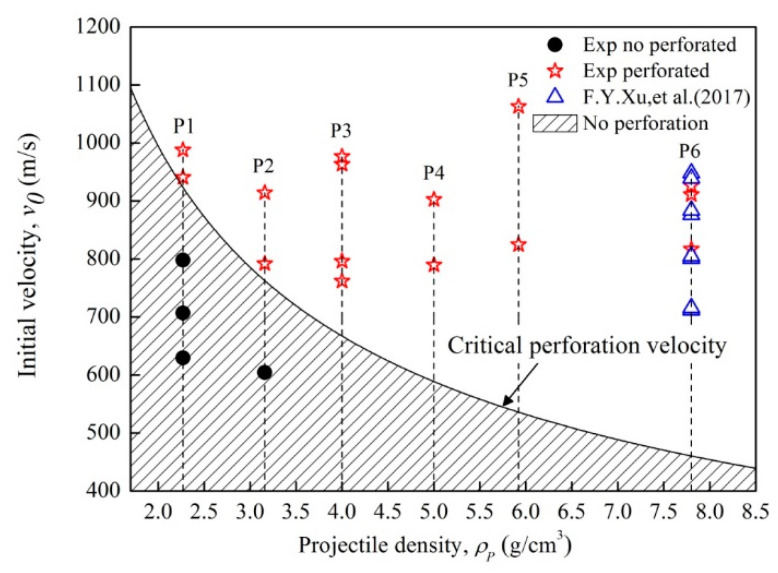
Critical perforation velocity of the reactive projectiles against the double-spaced plates.

Based on Equation (5), critical perforation velocity for different types of reactive projectiles can be obtained, as depicted in Figure 10. Meanwhile, experimental results regarding the perforation performance of different projectiles are also presented for comparison. As it shows, the critical perforation velocity decreases with the increase in projectile density, which indicates that the perforation capability improves. The area above the critical curve is for the projectiles which completely perforated the double-spaced plates, whereas the shadow area below the curve is for the projectiles which failed to perforate the targets. This indicates that the critical perforation curve provides effective prediction for the perforation performance of reactive projectiles with different densities. For example, at the initial velocity of 800 m/s, P1 projectile failed to perforate the rear plate and is under the critical curve in the shadow area, shown as the black dot marked in the figure. Whereas, other density reactive projectiles could completely perforate the plates and are above the curve. These are consistent with the results exhibited in Figure 7: the P1 projectile only produced a crack in the rear plate, but the other densities projectiles successfully perforated the double-spaced plates. Moreover, the experimental results of F. Y. Xu, et al. (2017) [23] are in agreement with the prediction curve, which also validates the correctness of the prediction and demonstrates tungsten is vital to improve the perforation performance of the PTFE/Al/W projectiles.

### 4.2. Perforation Diameter and Deflection Height of Front Plate

Dimensional analysis was developed to analyze the perforation diameter of the front plate. The parameters which affect the perforation diameter of the front plate include the reactive projectile parameters and the aluminum plate parameters. Parameters corresponding to the projectile include projectile diameter *d_p_*, projectile length *l_p_*, projectile initial velocity *v*_0_, and projectile density *ρ_p_*. The plate parameters include target density *ρ_t_*, target strength *σ_t_*, and target thickness *h_t_*. Despite the material compressibility, densities of the projectile and the aluminum plate are assumed to be constants. Moreover, the ignition delay time of reactive material was not considered due to the duration of delay time is extremely short [1]. Hence, the perforation diameter of the front aluminum plate *D_f_* is given by the following expression:

(6) $$D_{f}=f\left(d_{p},v_{0},\rho_{p},l_{p},h_{t},\rho_{t},\sigma_{t}\right)$$

Taking *d_p_*, *v*_0_, and *ρ_p_* as a unit system, then:

(7) $$\frac{D_{f}}{d_{p}}=f\left(\frac{l_{p}}{d_{p}},\frac{h_{t}}{d_{p}},\frac{\rho_{t}}{\rho_{p}},\frac{\sigma_{t}}{\rho_{p}v_{0}{}^{2}}\right)$$

From Equation (7), the dimensionless perforation diameter is related to the projectile geometry size, target thickness, material density, and strength. Since the projectiles’ shape is fixed, and only the thin plate (3 mm) is focused on in this study, in this case *l_p_*/*d_p_* and *h_t_*/*d_p_* are both constants, and the above formula could be simplified as:

(8) $$\frac{D_{f}}{d_{p}}=f\left(\frac{\rho_{t}}{\rho_{p}},\frac{\sigma_{t}}{\rho_{p}v_{0}{}^{2}}\right)$$

Referring to the existing empirical formulae for calculating the perforation diameter [24], the dimensionless perforation diameter is given by a power relationship as shown in Equation (9), where *C*, *α*, *β* are the constants to be determined:

(9) $$\frac{D_{f}}{d_{p}}=C{\left(\frac{\rho_{t}}{\rho_{p}}\right)}^{\alpha }{\left(\frac{\sigma_{t}}{\rho_{p}v_{0}{}^{2}}\right)}^{\beta }$$

Taking the natural logarithm of both sides of the above equation, Equation (9) can be rewritten as:

(10) $$ln\left(\frac{D_{f}}{d_{p}}\right)=lnC+\alpha ln\left(\frac{\rho_{t}}{\rho_{p}}\right)+\beta ln\left(\frac{\sigma_{t}}{\rho_{p}v_{0}{}^{2}}\right)$$

Combining the obtained experimental results, a multiple linear regression analysis is adopted on the above equation, where the strength of the aluminum plate is 470 MPa [22]. As such, the empirical formula for the dimensionless perforation diameter of the front aluminum plate is given as Equation (11):

(11) $$\frac{D_{f}}{d_{p}}=3.044{\left(\frac{\rho_{t}}{\rho_{p}}\right)}^{0.3756}{\left(\frac{\sigma_{t}}{\rho_{p}v_{0}{}^{2}}\right)}^{0.1707}$$

Observing the dimensionless relationship from Equation (11), the dimensionless perforation diameter of the front plate is related to the density ratio between the projectile and the target plate and the strength ratio of them, which demonstrates that material density and strength are dominant factors determining the perforation size.

**Figure 11:**
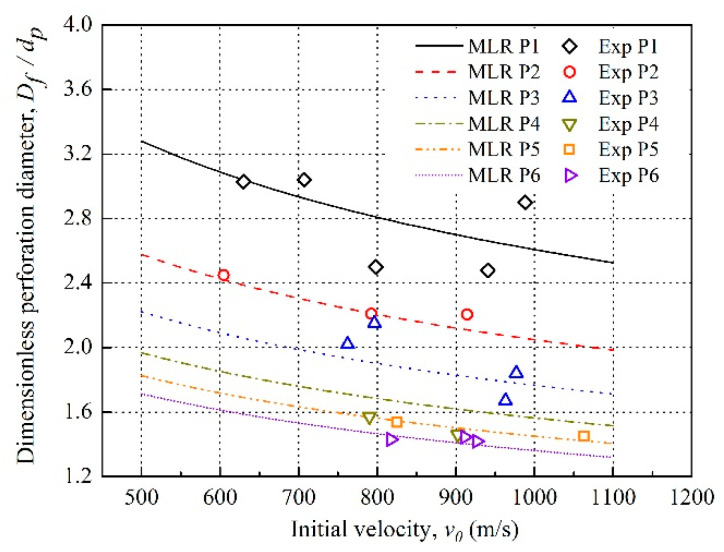
Dimensionless perforation diameter of the front plates versus initial velocity of the projectiles.

The multiple linear regression (MLR) curves of the dimensionless perforation diameter versus the initial velocity for different densities reactive projectiles are shown in Figure 11. It reveals that the dimensionless perforation diameter of the front aluminum plates decreases with the projectile initial velocity ranging from 500 to 1100 m/s. This results from two reasons: on the one hand, the increasing impact velocity causes the growth of kinetic energy and loading strain rate of the projectiles which enhance the dynamic response strength of the projectiles [5]; on the other hand, the higher initial velocity shortens the time for the projectiles to perforate the front plate, and then reduces the hole expansion effect of the reactive material deflagration reaction in the aluminum plate. As a result, the failure mode of the front plates is transformed from petalling failure to plugging failure, and the perforation diameter decreases.

**Figure 12:**
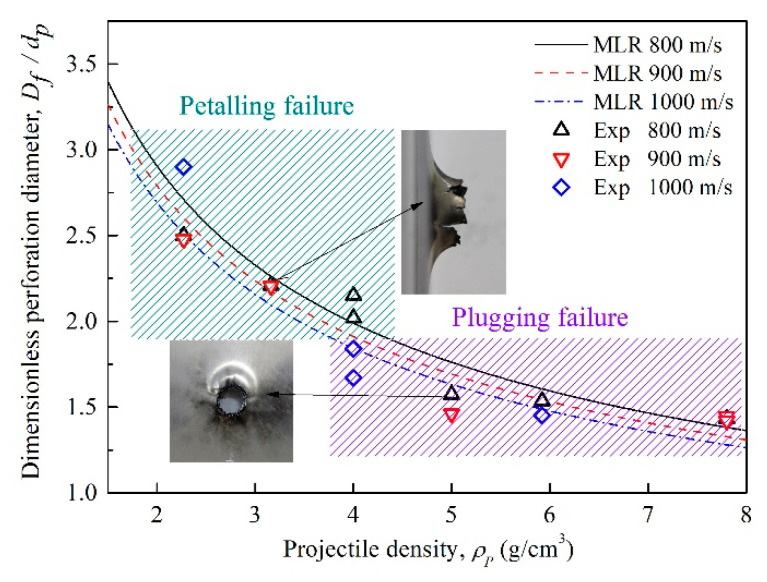
Dimensionless perforation diameter of the front plates versus the projectile density.

As the MLR curves and experiment data described in Figure 12, the dimensionless perforation diameters of the front plates decrease with the increasing projectile density at the same initial velocity. For the projectiles of a density lower than 4 g/cm^3^, due to the lower strength, less kinetic energy, and higher impact sensitivity characteristics [25], large deformation and violent deflagration occurred during perforating the plates, which make the front plate present petalling failure. Meanwhile, a longer perforation time enhances the deflagration expansion effect, and contributes to the perforation diameter for the low-density projectiles. In contrast, as the projectile density increases, the projectile strength and kinetic energy are enhanced while impact sensitivity becomes low due to the increased content of the inert tungsten. Dwell time for the perforation also decreases, which makes the deflagration expansion effect insignificant. Consequently, the perforation diameters of the front plates decrease and plugging failure exhibits.

**Figure 13:**
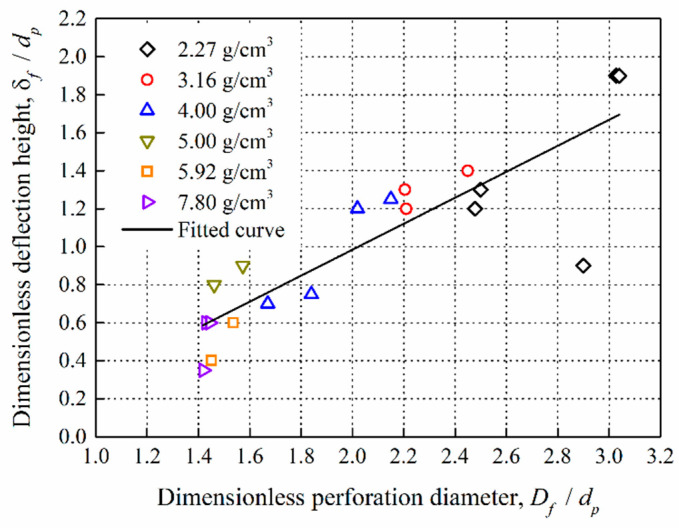
Dimensionless deflection height versus dimensionless perforation diameter.

Figure 13 depicts the dimensionless relationship between the deflection height and the perforation diameter of the front plates. For the aluminum plates which were impacted by the reactive projectiles, the deflection height is proportional to the perforation diameter. The fitted curve between the dimensionless deflection height and the perforation diameter of the front plates is shown in Equation (12):

(12) $$\frac{\delta_{f}}{d_{P}}=0.6835\frac{D_{f}}{d_{P}}-0.3828$$

where *δ_f_* (mm), *D_f_* (mm), *D_p_* (mm) are the perforation deflection height, perforation diameter, and projectile diameter, respectively. It reveals that there is a positive correlation between them, and the perforation deflection height can then be estimated according to the equation.

### 4.3. Damage Behavior of Rear Plate

**Figure 14:**
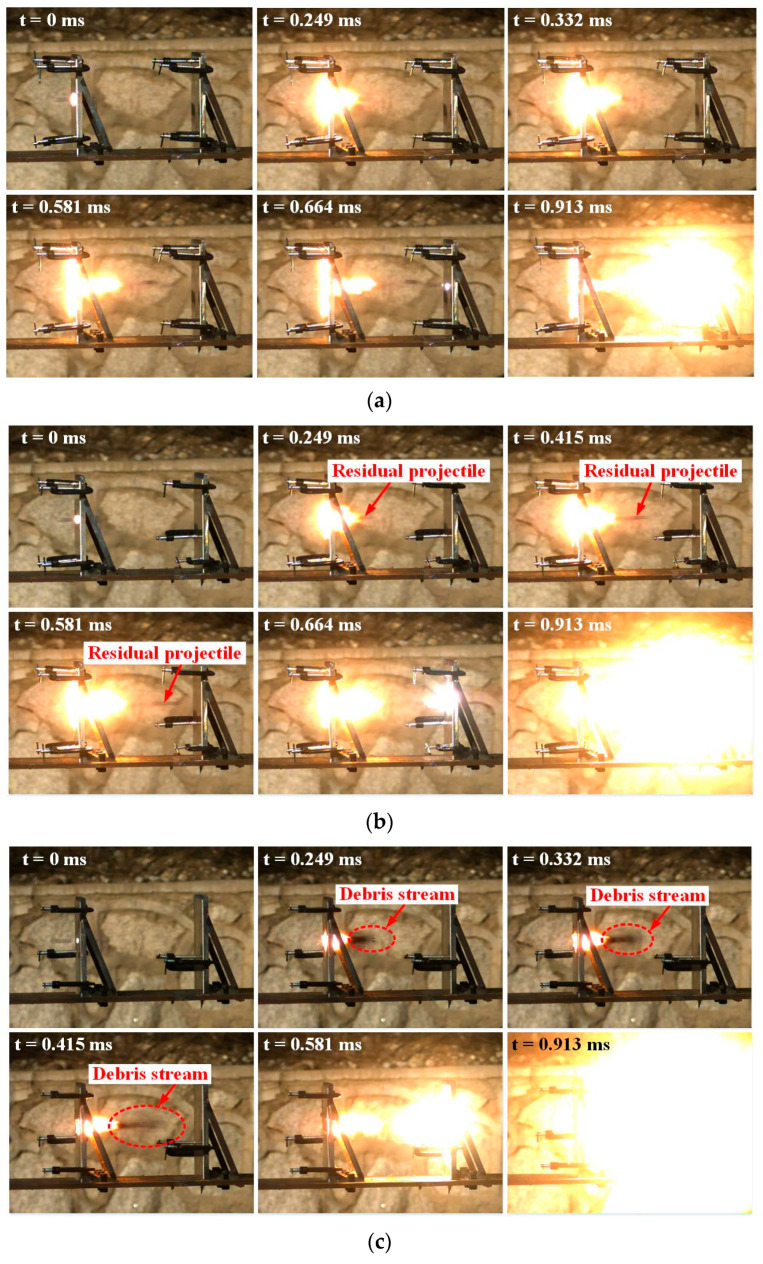
High-speed video frames of the reactive projectiles perforating double-spaced aluminum plates: (**a**) P1 at 798 m/s, (**b**) P3 at 762 m/s, (**c**) P6 at 817 m/s.

Figure 14a–c show high speed video sequences of three types of reactive projectiles perforating the double-spaced aluminum plates, respectively, in which P6 is the highest density followed by P3 and P1. The initial velocity of the projectiles is about 800 m/s. As Figure 14 shows, at 0.581 ms, P1 projectile has not hit the rear plate, while P6 projectile has already impacted the rear plate and reacted. This indicates the residual velocity of high-density projectile after perforating the front plate is higher than that of the low-density one.

The flame distribution for the front plates reveals that the P1 projectile flame is mainly distributed in front of the front plate. The flame radial distribution area on the plate surface is wider than that of the other types of projectiles as Figure 14 depicted. This is due to the high content PTFE matrix for the P1 projectile, which results in the characteristics of low dynamic strength and high sensitivity [5,25]. The projectile is easier to deform and react when it impacts the target plate, and the flame spreads a broader distribution area on the surface of the front plate. However, with the increasing tungsten content of the projectiles, the density and strength for P3 projectile improve, but the impact sensitivity falls. These factors reduce the compressibility of the projectiles, narrow the flame radial distribution area on the plate surface, and shorten the time for the projectiles perforating the front plates. The flame concentration region moves backwards from the front to the back of the plate, as shown in Figure 14b. When the tungsten mass fraction of the projectiles becomes the highest, P6 projectile reaches the maximum density, with the highest strength, the lowest impact sensitivity, and chemical energy. As depicted in Figure 14c, the P6 projectile produces the smallest flame region during perforating the front plate. The flame region is behind the front plate, and no flame is distributed in front of the front plate.

As arrowed in Figure 14b,c, the P3 projectile forms a residual penetrator after perforating the front plate, whereas the P6 projectile produces a debris stream behind the front plate due to the brittleness of the high-density projectile. The debris stream is composed of small fragments of reactive projectile, and expands from 0.249 to 0.415 ms, which enlarges the contact area of the reactive fragments and rear plate. Comparing the spherical flame region at 0.913 ms, the chemical reaction of P6 is much more violent, and the flame region around the rear plate is the largest, followed by P3 and P1.

**Figure 15:**
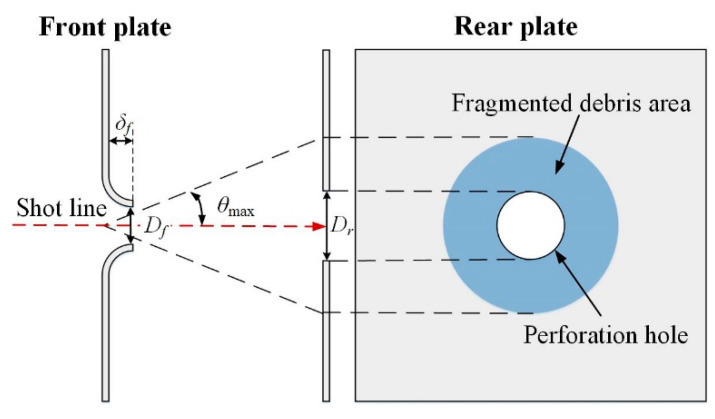
Perforation model for the double-spaced plates.

The damage mechanisms of the low-density and high-density reactive projectiles to the rear plates are different, which leads to differences in the damage behavior of the witness plates. Figure 15 depicts a diagram of the perforation model for the double-spaced plates, in which *D_f_*, *D_r_* are the perforation diameter of the front plate and rear plate, respectively. *δ_f_* is the deflection height of the front plate. For the high-density reactive projectile, as depicted in the diagram, an annular debris concentration area is around the perforation hole on the rear plate, which is the significant difference between the high-density reactive projectiles and the low-density ones in damage behavior of rear plates. Taking the initial collision point between the front plate and the projectile as the vertex, the angle between shot line and outer edge of the annular debris concentration area in the rear plate is defined as the maximum spray angle of the projectile debris stream *θ*_max_.

**Figure 16:**
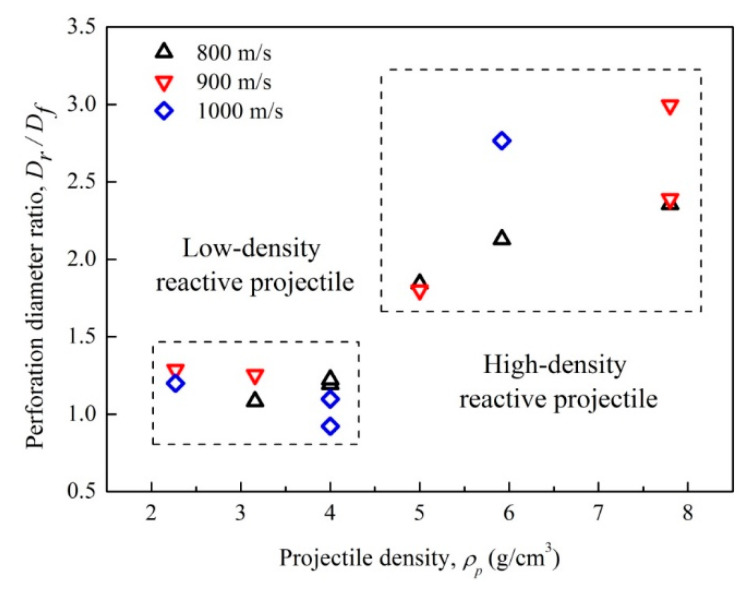
Perforation diameter ratio of rear plate to front plate versus projectile density.

The test data of the perforation diameter ratio for the rear plate to the front plate of various densities projectiles under different initial velocities are shown in Figure 16. When projectile density is lower than 4.0 g/cm^3^, the perforation diameter ratio is mainly between 1.0 and 1.5, and the rear plate perforation size is almost equal to that in the front plate. The rich content of the PTFE matrix makes the low-density projectile compressible and less fragmented during perforation, so the projectile is relatively complete with no significant fragmentation after perforating the front plate. However, for the high-density projectiles of densities higher than 4.0 g/cm^3^, the perforation diameter ratio is increased significantly with the projectile density. This is due to that the mass fraction of tungsten being greater than 60%, so the kinetic energy and brittleness of the projectiles are obviously enhanced with the increasing tungsten content. Meanwhile, as a binder, the reduction of the PTFE matrix weakens the connection between metal particles, and makes the projectile more easily fragmented as it impacted with the plate. The debris stream generates behind the first plate, and impacts the witness plate; therefore, the perforation size of the rear plate is enlarged, and the perforation diameter ratio increases.

**Figure 17:**
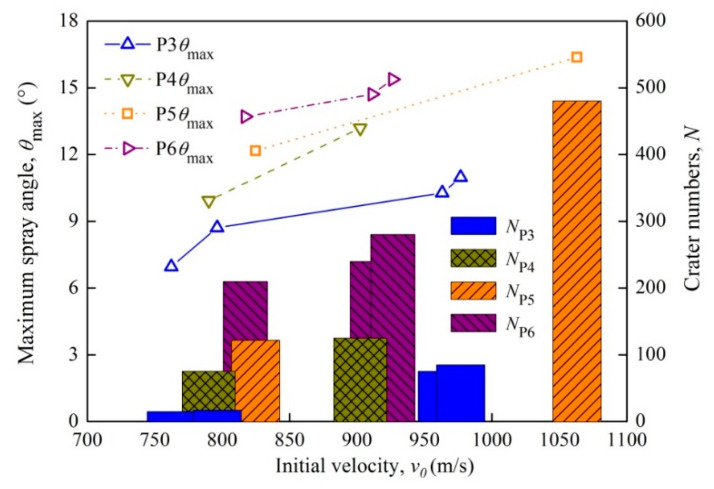
Maximum spray angle of the projectile streams and crater numbers of the concentration debris area versus projectile initial velocity.

The debris streams’ maximum spray angle *θ*_max_ and the crater numbers *N* of the debris concentration area in the rear plates for the P3–P6 projectiles are displayed in Figure 17. It shows that the maximum spray angle of the debris stream and the crater numbers in the rear plate grow with the increasing projectile density and initial velocity. This indicates that the projectiles become more fragmented and produce more debris. Thus, the contact area of the projectile debris stream and rear plate expands, which enlarges the perforation diameter and brings prominent damage to the rear plates.

## 5. Conclusions

Experiments and theoretical analysis were conducted to study the perforation behavior of double-spaced thin aluminum plates by PTFE/Al/W reactive projectiles of densities ranging from 2.27 to 7.80 g/cm^3^. The main conclusions drawn from the analysis are as follows:

(1) Critical perforation velocities for reactive projectiles perforating the double-spaced plates were obtained. With the projectile density increasing, the critical perforation velocity decreases. It shows that tungsten plays vital role in improving the PTFE/Al/W projectile perforation performance.

(2) For the perforation characteristics of the front plates under the same initial velocity, the failure mode of the perforation hole transforms from petalling failure to plugging failure as the projectile density increases. Meanwhile, the perforation diameter decreases with increasing projectile density and initial velocity. The predicted dimensionless perforation diameter and deflection height of the front plate was obtained through dimensional analysis, which fit well with the experimental results. It shows that density and strength of the projectile and target plate are dominant factors affecting the perforation size. In addition, there is a positive relationship between the deflection height and perforation diameter of the front plate.

(3) The destruction behavior of low-density and high-density reactive projectiles to the rear plates are different. For the low-density ones, the perforation diameter ratio of rear plate to front plate is between 1.0 and 1.5. However, as the projectile density is higher than 4 g/cm^3^, the perforation diameter ratio increases significantly with the projectile density and initial velocity. Meanwhile, high-density projectiles produce a debris stream after perforating through the front plates, which enlarges the perforation size of the rear plates, and forms an annular debris concentration area around the perforation hole. The debris stream maximum spray angle and the crater numbers in the debris concentration zone both increase with the projectile density and initial velocity.

## Figures and Tables

**Table 1:** Basic parameters of reactive material projectiles.

Type	Component Mass Ratio (wt.%)			Density (g/cm^3^)	Mass (g)
	PTFE	Al	W		
P1	73.50	26.50	0.00	2.27	2.13
P2	50.16	18.08	31.76	3.16	3.00
P3	37.49	13.51	49.00	4.00	3.78
P4	28.03	10.11	61.86	5.00	4.70
P5	22.20	8.00	69.80	5.92	5.59
P6	14.46	5.22	80.32	7.80	7.35

**Table 2:** Size and density of the projectiles.

Projectile No.	Diameter (mm)	Height (mm)	Mass (g)	Actual Density (g/cm^3^)
P1-1	9.96	12.08	2.13	2.26
P1-2	10.00	12.01	2.13	2.26
P1-3	10.00	12.06	2.14	2.26
P1-4	9.98	12.03	2.14	2.27
P1-5	9.97	12.07	2.15	2.28
P2-1	10.00	12.01	3.00	3.18
P2-2	9.99	12.03	3.00	3.18
P2-3	10.00	12.02	2.99	3.17
P3-1	9.97	12.06	3.78	4.01
P3-2	10.00	12.07	3.77	3.98
P3-3	9.96	12.05	3.78	4.03
P3-4	10.00	12.05	3.77	3.98
P4-1	10.00	12.00	4.71	5.00
P4-2	9.98	12.02	4.71	5.01
P5-1	10.00	12.03	5.59	5.92
P5-2	9.99	12.01	5.59	5.94
P6-1	9.98	12.02	7.35	7.82
P6-2	9.98	12.03	7.33	7.79
P6-3	10.00	12.03	7.35	7.78

**Table 3:** Experimental results.

Projectile No.	Initial Velocity (m/s)	Perforation Diameter of Front Plate (mm)	Deflection Height of Front Plate (mm)	Equivalent Perforation of Rear Plate (mm)
P1-1	629.8	Φ30.28	19	No perforated
P1-2	707.0	Φ30.40	19	No perforated
P1-3	798.1	Φ25.00	13	No perforated
P1-4	940.9	Φ24.78	12	Φ31.88
P1-5	988.1	Φ29.00	9	Φ34.78
P2-1	604.6	Φ24.50	14	No perforated
P2-2	792.3	Φ22.10	12	Φ23.94
P2-3	914.3	Φ22.05	13	Φ27.64
P3-1	762.2	Φ20.20	12	Φ24.10
P3-2	796.3	Φ21.50	12.5	Φ26.32
P3-3	963.2	Φ16.70	7	Φ15.40
P3-4	976.8	Φ18.40	7.5	Φ20.19
P4-1	790.0	Φ15.73	9	Φ28.99
P4-2	902.3	Φ14.61	8	Φ26.32
P5-1	824.7	Φ15.36	6	Φ32.70
P5-2	1063.1	Φ14.51	4	Φ40.13
P6-1	817.2	Φ14.30	6	Φ33.68
P6-2	911.2	Φ14.45	6	Φ34.52
P6-3	926.4	Φ14.19	3.5	Φ42.50
